# Building sustainability assessment: A comparison between ITACA, DGNB, HQE and SBTool alignment with the European Green Deal

**DOI:** 10.1016/j.heliyon.2024.e34478

**Published:** 2024-07-10

**Authors:** Dorra Kouka, Mariateresa Russo, Francesco Barreca

**Affiliations:** Mediterranean University of Reggio Calabria, Department of Agriculture, Località Feo Di Vito, 89122, Reggio Calabria, Italy

**Keywords:** Green building assessment tools, Sustainability, European Green Deal, Multi-criteria decision making, Boolean analysis, Fuzzy logic analysis

## Abstract

The environmental impact of the construction industry is very heavy. Therefore, the global community is further developing green building assessment tools in order to enhance their efficiency in matching sustainability goals and being more environmentally friendly. An analysis approach by means of Multi-Criteria Decision Making (MCDM) was carried out to examine the degree of response of different green building tools utilized in Europe, namely (Innovazione e Trasparenza degli Appalti e la Compatibilità Ambientale ITACA, Deutsches Guetsiegel Nachhaltiges Bauen (DGNB), Haute Qualité Environnementale (HQE) and Sustainable Building Tool (SBTool), to the eight criteria of the European Green Deal (EGD), a roadmap elaborated by the European Commission to enhance sustainability deployment in the region. The first phase of the analysis consisted of a Boolean MCDM aiming to define to which criterion of the EGD each indicator in the tools checklists is linked. These data obtained were later examined by means of Fuzzy Logic to obtain comparable results showing how much each tool helps more following the European roadmap towards sustainability. This work intends to compare the efficiency of the most used tools in Europe for building sustainability evaluation while being based on a particular specified reference and not only the sustainable goals in general. This work also shows the efficiency of combining two MCDCM techniques to obtain better analyzing output. The result of this study shows that the DGNB is the most effective method for connecting all EGD criteria in a balanced manner. The HQE tool demonstrated a strong ability to effectively integrate the objectives of the EGD, except for the energy evaluation aspect. For the ITACA tool, it closely aligned DGNB in its response to the EGD, although it had an absent focus on the smart and sustainable shift of mobility. SBTool demonstrated average performance when compared to other protocols. This was expected since SBTool was the basis on which ITACA, DGNB, and HQE were constructed.

## Abbreviation definition

BMCDM: Boolean Multi Criteria Decision Making
BRE: Building Research Establishment
BREEAM: Building Research Establishment Environmental Assessment Method of Great Britain
CASBEE: Comprehensive Assessment System for Built Environment Efficiency
DGNB: Deutsches Guetsiegel Nachhaltiges Bauen
EGD: European Green Deal
FAO: Food and Agriculture Organization
France GBC: France Green Building Council
GBC: Green Building Challenge
GBTool: Green building tool
HQE: Haute Qualité Environnementale
iiSBE: International Initiative for a Sustainable Built Environment
ITACA: Innovazione e Trasparenza degli Appalti e la Compatibilità Ambientale
LEED: Leadership in Energy and Environmental Design
MADM: Multi-Attribute Decision Methods
MCDM: Multi Criteria Decision Making
MODM: Multi-Objective Decision Methods
SBC: Sustainable Building Council
SBTool: Sustainable Building Tool
USGBC: United States Green Building Council

## 1 Introduction

The global community has been thinking about sustainability and quality in all areas ever since significant threats became obvious to our green planet, such as pollution, water scarcity, climate change, food insecurity, and so forth [1]. Consequently, it has been mandatory to ascertain the sources of these concerns. Then, the infrastructure and buildings are particularly important to the growth of both economies and societies, and it has been reported that they, undoubtedly, use a lot of resources and energy [2]. This phenomenon is being driven by the ongoing population growth and the increasing demand for constructing several types of buildings, including residential, educational, industrial, and others. Precisely, In October 2022, there was a 1.3 % increase in building construction in the Euro area compared to September 2022 [3], while knowing that this industry continues to account for 36 % of the greenhouse gas emissions related to energy, and 40 % of total energy consumed in Europe [4]. Despite a significant rise of awareness and global progress in reducing the impact of construction in our globe, the 2022 buildings-GSR reported that the sector's overall energy consumption and CO_2_ emissions grew in 2021 in comparison to pre-pandemic levels. From 2020 to 2021, building energy demand has increased by 4 percent and CO_2_ emissions by five percent [5]. In addition, the construction industry is responsible for producing 25 % of global solid waste, which primarily consists of materials that are difficult to reuse. Indeed, solid waste is an unavoidable byproduct in the life cycle of structures, but workers often dispose of these wastes carelessly and in unsuitable locations [[6], [7], [8]]. Rather than having an impact on the environment, there are buildings that have a direct connection with food. This specific kind of building must perform efficiently, respecting food safety and security rules. Otherwise, it could be an obvious cause of food loss and a health threat for consumers. In reality, this task is challenging due to the diverse range of sizes and structures of buildings related to this field such as warehouses, industries and retails. Each sort of these facilities doesn't have a standardized structure and design. It is evident that the performance varies depending on the specific types of products being carried. Failure to satisfy storage conditions and criteria puts the goods at danger of spoilage or being sold to customers in a deteriorated state [9]. In this context, The Food and Agriculture Organization (FAO)of the United Nations reported in 2015 [10] that in China, approximately 120 billion kg of the nation's grain storage capacity at the enterprise level is allocated to antiquated and hazardous warehouses with substandard conditions. This leads to an annual loss of more than 7.5 billion kilograms. Moreover, in 2017, the FAO gave an account that one-third of produced food is lost because of bad storage conditions in carrying buildings besides other reasons throughout the entire supply chain [11]. Recently, the Food Waste Index Report of the United Nations mentioned that food waste in retails reached 13 % in 2019 [12]. These considerations have encouraged scientists and authorities to explore methods for evaluating facilities and their performances.

Building evaluation may be linked to sustainability assessment tools or it may simply refer to any method or technique used to enhance a structure's functionality with respect to any metric. Developed within the principle of sustainability, which was defined during the Rio Earth Summit in 1992 as “Meeting the needs of the present generation without compromising the ability of future generations to meet their own needs” [13], the building assessment tools were created with the future in mind. In other words, trying to locate an equilibrium between the usually conflicting goals of environmental, economic, and social aspects [14]. The elaboration of these tools made a huge advancement in the construction industry and the architecture of modern buildings. The main objective of Sustainable building Assessment (SBA) tools is to demonstrate the evolution of the structure over time [15]. However, also they served as a reference for the decision-making process throughout the design phase, where the future performance of the building would be analyzed and would likely yield positive results [[16], [17], [18]]. Buildings that are certified by these rating systems are generally recognized as being more energy-efficient, offering a healthier living environment, economically effective, and having positive impact on the property's general reputation [19]. Numerous building rating systems already exist on a global scale, and their numbers are only going to grow in the future. These different tools were developed to evaluate many aspects of sustainability, primarily environmental, using methods based on the judgment of experts in the field or specifically qualified individuals [20]. They are also devoted to forecasting the structures' evolution in the following several decades. Moreover, they serve as a support for decision making in the world of construction [[21], [22], [23]]. The following are the most well-known tools; Since 1988, the Building Research Establishment (BRE) has been developing the BREEAM (Building Research Establishment Environmental Assessment Method of Great Britain) [24], the first technique for assessing the performance of green buildings ^2526^. The United States Green Building Council (USGBC) created the Leadership in Energy and Environmental Design (LEED) [27] program in 1998 [28]. Currently, the LEED certification method is widely acknowledged as the predominant standard for assessing the environmental sustainability of buildings [27,29]. The Comprehensive Assessment System for Built Environment Efficiency (CASBEE) [30] was initially conceived in 2001 as an architectural design tool through a collaborative effort between industry, government, and academia in Japan. CASBEE is a widely utilized green building rating system and serves as the standard for the Sustainable Building Reporting System for Japan [25,31]. Since 1996, A group consisting of more than twelve nations has been collaborating on the development of the Green Building Challenge (GBC) evaluation methodology, with GBTool serving as the corresponding software application. Natural Resources Canada initiated the GBC process, but in 2002, they passed the baton to the International Initiative for a Sustainable Built Environment (iiSBE). Before applying it to case study structures, national teams customize the standard software to suit their specific circumstances [[32], [33], [34]]. For the tools used in the European level, In France, the HQE (Haute Qualité Environnementale) was established in 1996 with public authority support. It was later fused in 2016 with the France Green Building Council (France GBC) association, to form the Alliance HQE-GBC [35,36]. In Italy, the Sustainable Building Council (SBC) organization developed the “Innovazione e Trasparenza degli Appalti e la Compatibilità Ambientale” ITACA building environmental categorization, allowing for the evaluation of various planned applications throughout the life cycle of structures. Its use is possible at many building stages, including each phase of construction, refurbishment, and operation [26,37]. On the other hand, “Deutsches Guetsiegel Nachhaltiges Bauen » (DGNB) is the assessment tool adopted in Germany which was established by the German Ministry of Transport, Construction, and Urban Planning (BMVBS) and the German Sustainable Building Council [[38], [39], [40]].

The efficacy of these various tools has been examined in numerous research reviews proving the importance of this topic and the rising awareness about the necessity of assessing buildings. The objective is to delineate the strengths and weaknesses of the tools [25,26,31,40,41]. Most of the studies are in the form of reviews and critical analysis on how they assume these protocols are performing based on sustainability pillars. On the other hand, some other studies opted to establish a goal reference to base on their comparison. However, there is no research that has taken the European Green Deal as objective, or has chosen the ITACA, DGNB, HQE, and SBTool for confrontation as they derive from the same origin and are developed and applied in the same region. In fact, most of the consulted works include LEED and BREEAM. Fei, Wenmei et al. (2021) [42] chose the sustainability goals as a reference to compare the different tools, while Bernardi, Elena et al. (2017) [43] focused on the environmental impact assessment in the tools’ checklists. In another study, Mattoni, B. et al. [26] conducted a differential analysis mentioning the importance of a tool being developed and used within the same country or not. These references helped choose the concept of this study that takes into consideration the location where the selected tools are utilized, and the orientation of the countries developers of these tools from sustainability perspective. The European building evaluation methods ITACA, DGNB, and HQE, utilized in Italy, Germany, and France respectively, were chosen for analysis in this study. In addition, the SBTool was also joined since it constituted the foundation for the abovementioned tools, and because of its current use in Portugal. The target is to comprehensively evaluate the performance of various European sustainability buildings assessment tools, using a European sustainability framework as a reference.

This evaluation aims to identify opportunities for enhancing sustainability practices within the construction sector across the region. By systematically comparing these tools, we seek to uncover strengths and weaknesses in their methodologies and applications through their checklists and the indicators they suggest, thereby facilitating informed improvements in sustainability assessments. Such comparative analysis is crucial for refining the evaluation processes of buildings, ultimately contributing to the broader goal of advancing sustainable construction practices in Europe. This framework is the European Green Deal (EGD); the closer the alignment of the tools' indicators with the criteria of the EGD, the more effective the tools are considered to be.

**Fig. 1:**
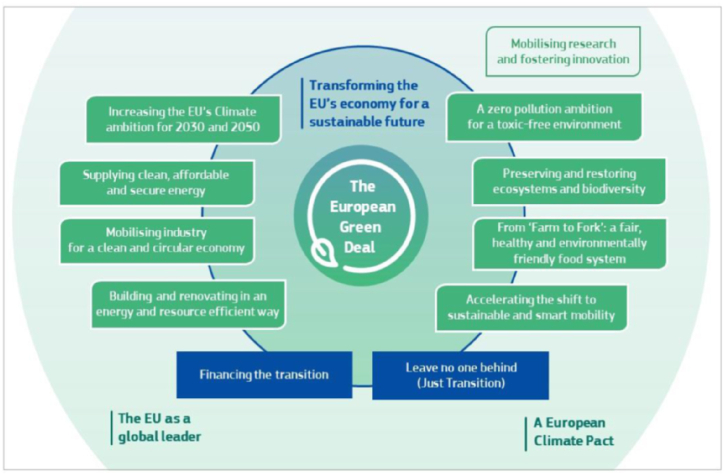
The European Green Deal criteria. (For interpretation of the references to colour in this figure legend, the reader is referred to the Web version of this article.)

An overview of the EGD and its eight criteria are summarized in this work in order to better understand the methodology furthermore. Actually, the effects of climate change and ecological degradation pose a significant and fundamental threat to the entire planet including Europe. Since this topic has consistently been a cause of concern for the European Commission, the committee has embraced the goal of lowering net greenhouse gas emissions by a minimum of 55 % by the year 2030 and reaching neutrality by 2050. The “European Green Deal” (EGD) is the term given to this project that was introduced amidst the ongoing global COVID-19 pandemic. With a defined set of objectives, the aim is to enhance energy efficiency, optimize transportation, minimize pollution, ensure access to healthy and high-quality food, and effectively incorporate the principles of a circular economy [44]. The pillars of the European Green Deal were established as eight criteria [45], which are as follows (Fig. 1):

1 Increasing the EU's climate ambition for 2030 and 2050: When compared to the levels that existed in 1990, the European Council announced that it will lift the climate target for the European Union to at least 55 % net reductions in greenhouse gas emissions by the year 2030 [44]. It involves establishing more stringent regulations and benchmarks for every activity that contributes to the release of greenhouse gases, with the aim of achieving this objective.
2 Supplying clean, affordable, secure energy: 75 % of greenhouse gas emissions in the European Union come from using and making energy. In order to achieve a shift towards a sustainable energy system, the European Commission has established a number of objectives. These include revising policies and frameworks and expanding the use of clean energy sources. In simple terms, the objective of the renewable energy directive is to enhance the proportion of renewable energy in the European Union's economic system [46].
3 Mobilizing industry for a clean and circular economy: The focus is on promoting the creation of environmentally friendly products and implementing cyclic manufacturing methods. Furthermore, it aims to involve customers in this approach by increasing their awareness about the lifespan, use, and repair techniques of the products they buy. The strategy targets particular sectors like electronics, furniture, and high-impact intermediary products industries to better include circularity in their production processes while also decreasing waste rates [47].
4 Building and renovating in an energy and resource-efficient way: This strategy aims to enhance the performance of the currently inefficient European building stock. by promoting their rehabilitation. The purpose of this action is to enhance energy and resource efficiency, hence improving the well-being of the individuals occupying the buildings [44].
5 A zero-pollution ambition for a toxic-free environment: The strategy attempts to achieve an improved environment, free from detrimental chemicals. The objective is to motivate chemical industries to compete in replacing toxic chemicals with safer alternatives and gradually phasing out the usage of the most dangerous compounds for non-essential reasons [48].
6 Preserving and restoring ecosystems and biodiversity: The objective is to safeguard and rehabilitate the biodiversity and efficient ecosystems of Europe, as they are crucial for enhancing the EU economy and societies' ability to withstand future challenges, such as the effects of climate change, forest fires, food scarcity, and disease outbreaks [49].
7 Farm to Fork: a fair, healthy, and environmentally friendly food system: The principal goals of the approach are to ensure a sufficient provision of nutritious food at an affordable cost, while staying within the boundaries of our planet's resources. The strategy promotes healthier dietary habits by encouraging sustainable agriculture, farming, and food production [50].
8 Accelerating the shift to sustainable and smart mobility: Since transportation is responsible for 25 % of the greenhouse gas emissions in the European Union, this strategy promotes the advancement of transportation networks to enhance interconnectivity across regions in Europe, while additionally reducing carbon emissions in the sector by reducing the reliance on fossil fuels in both freight and passenger transport inside the EU [51].

**Fig. 2:**
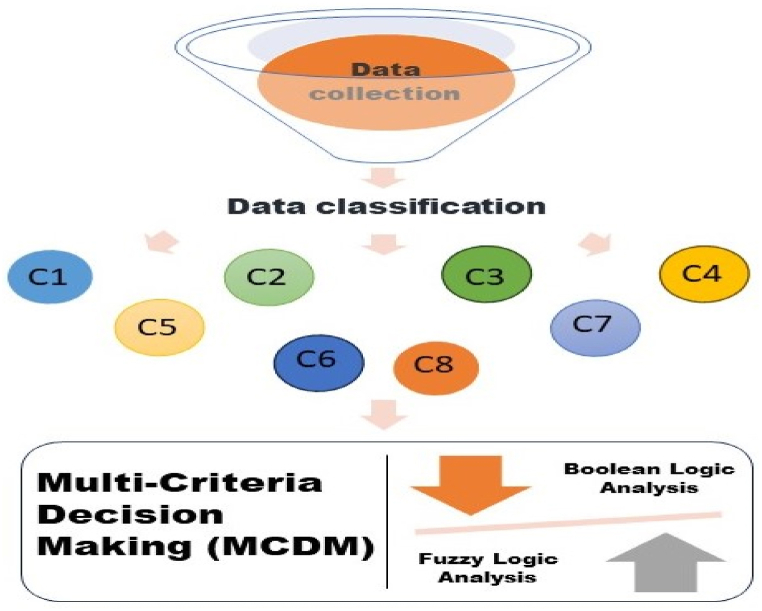
Methodology diagram.

**Fig. 3:**
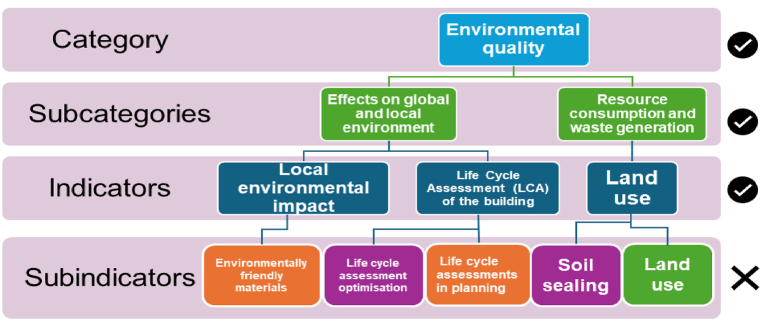
Diagram presenting the process of selecting the tools checklists' indicators. The signs indicate that the methodology is limited to the indicators level.

**Fig. 4:**
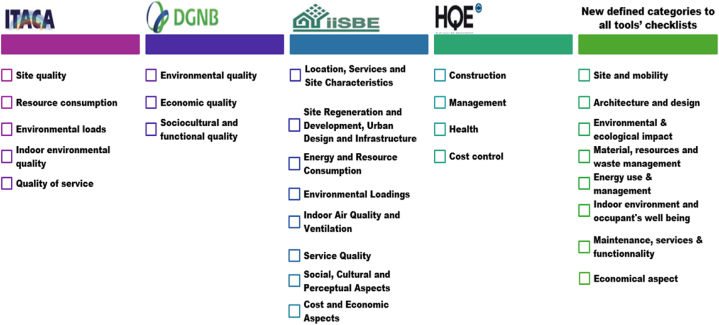
Original categories of each green building tool and the new defined categories adopted in the methodology. (For interpretation of the references to colour in this figure legend, the reader is referred to the Web version of this article.)

## 2 Material and methods

The study's methodology is summarized in Fig. 2. In more details, the different steps are described as follows:

- **Data collection:** This phase involves collecting the data. The data are the checklists of each tool, including the different indicators that need to be assessed throughout the building evaluation (Co_2_ emissions, ventilation, daylight factor, etc.). All chosen checklists are for new constructions and were obtained directly from the websites of the tools' developers.

Due to the large volume of data, the sub-indicators were excluded and only the primary indicators were considered. A small sample example is illustrated in Fig. 3.

- **Data categorization:** The indicators of the tools are originally classified into separate categories based on their connection to energy, environment, economic factors, water, etc. However, from one tool to another, the same indicator might be named and classified differently. As an example, the indicator “potable water for indoor uses” belongs to resource consumption in ITACA but for DGNB is in the “environmental quality” category. To address this limitation, eight clusters were implemented depending on their independence and the correlation between the indicators they include (Fig. 4).

The new clusters are:

1 Site selection and mobility (Site characteristics, site choice, accessibility, transport …)
2 Architecture and design (Technological, aesthetic, and creativity aspects)
3 Energy use and management (Energy consumption, management, production …)
4 Material and resources management and waste management (Material use, material characteristics, material choice, resources exploitation, management of resources and material …)
5 Environmental and ecological impact (emissions, pollution, impacts on ecosystems, life cycle assessment …)
6 Indoor environment and occupant's well-being (Occupants' comfort, Indoor air quality, hygiene, temperature …)
7 Maintenance, services & functionality (maintenance activities, controllability, Renovation …)
8 Economical aspect (Costs, costs management …)

In subsequent sections of this study, this newly generated data set was used as the basis for evaluating the different green tools.

- **Data treatment and analysis:** The analysis of how much the indicators help the tool to fully evaluate all building performances and sustainability was chosen to be realized through multi-criteria-decision-making (MCDM) techniques. In this step, to make sure the use of MCDM is efficient, two analysis methods were followed: a Boolean Multi Criteria Decision Making (BMCDM) analysis, and a fuzzy logic analysis. As the different considered tools are used in the European Union, The European Green Deal (EGD) project was selected as a reference to base this analysis on.

The Boolean technique examines the tool's checklists from the very particular to the general (from indicators to categories to finally the entire tool). This is to assess the correlation between each single indicator and every EGD criterion. In fact, each indicator contributes, with a certain degree, in building the sustainability of the tool. Therefore, it is very important to each indicator solemnly. However, the Boolean analysis does not consider the uncertainty and the ambiguity related to the authors' judgement. Accordingly, the utilization of the fuzzy logic technique comes to fill this gap by including in the analysis the indicator's influence and importance within the tools' checklists. Consequently, the combination of these two techniques allows to pick the best tool to meet the EGD strategy.

### 2.1 Multi-criteria-decision-making

The MCDM methodology has been extensively employed in research to prioritize, categorize, or choose alternatives when decision-making is complex and susceptible to being influenced by many factors [52,53]. There are three essential steps for MCDM consisting of: criteria identification, weights definition, selection of the convenient MCDM method [54]. In other words, a set of alternatives A_1-n_ are differently responding to a set of criteria C_1-m_, to each a weight w_1-m_ is attributed. The answer of A_1-n_ to C_1-m_ is a variable x_1-nm_^53^. The matrix of these components is shown in Table 1.

**Table 1:** MCDM (Multi-Criteria-Decision-Making) matrix model.

	C_1_	C_2_	…	C_m_
A_1_	W_1_X_11_	W_2_X_12_	…	W_m_X_1m_
A_2_	W_1_X_21_	W_1_X_22_	…	W_m_X_2m_
…	…	…	…	…
A_n-1_	W_1_X_n-11_	W_2_X_n-12_	…	W_m_X_n-1m_
A_n_	W_1_Xn_1_	W_2_X_n2_	…	W_m_X_nm_

**Fig. 5:**
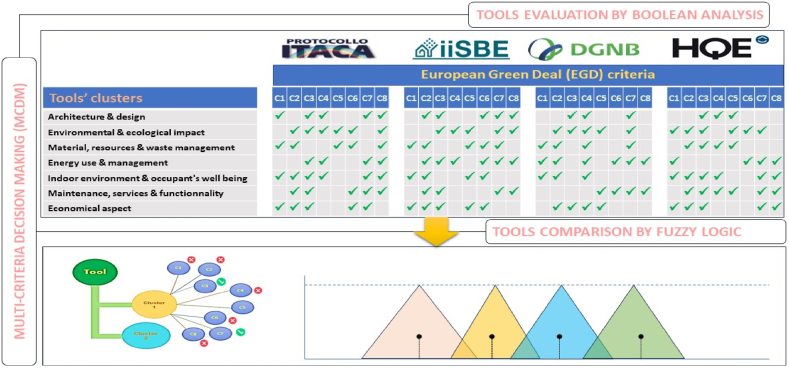
Diagram representing the techniques (Boolean Analysis and Fuzzy Logic Analysis) of MCDM used in the methodology.

MCDM is divided in two major methods; the Multi-Objective Decision Methods (MODM) which serves to solve mathematical programming and design problems, and the Multi-Attribute Decision Methods (MADM) which serves for reducing the number of alternatives by selecting the best options [55]. These two methodologies are further divided into numerous techniques, of which two were chosen to conduct the assessment of the tools: the Boolean logic (BMCDM) and the Integrated fuzzy AHP technique [56] (Fig. 5).

### 2.2 Boolean logic analysis

**Fig. 6:**
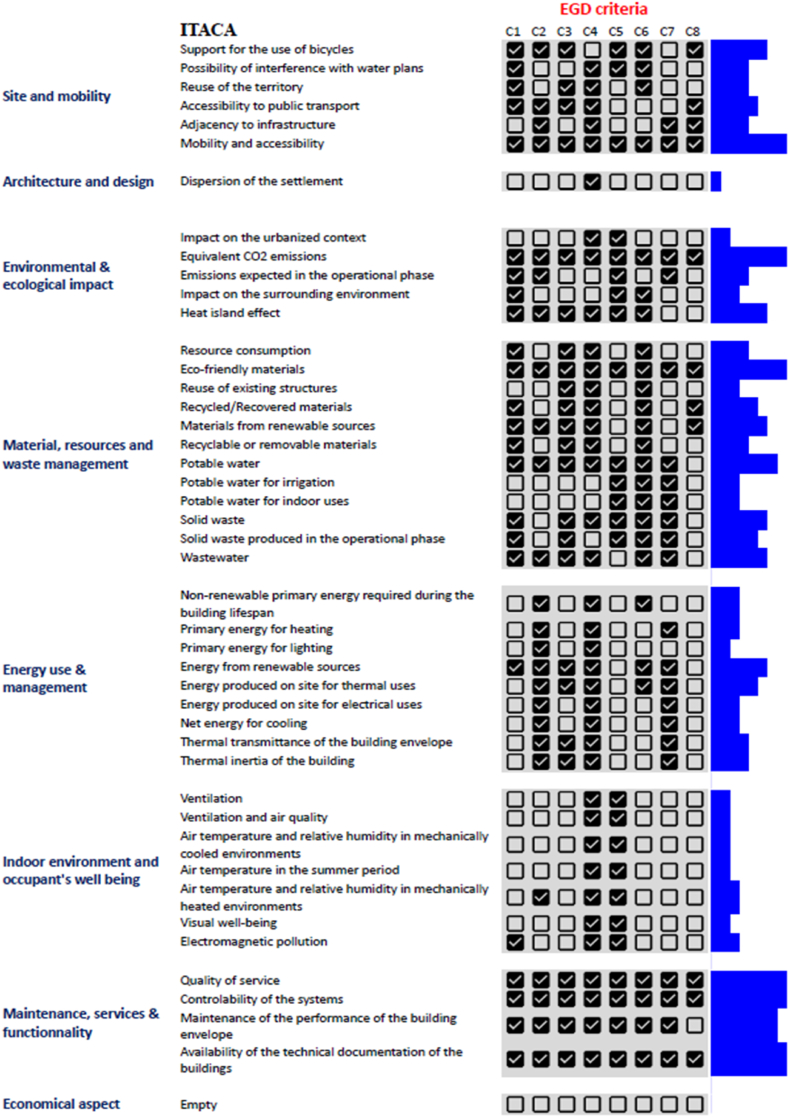
ITACA checklist (categories and indicators on the left), the grid presenting the response to each indicator to the EGD criteria (the middle), diagrams presenting in vertical histograms the level of response to all EGD criteria.

The Boolean method is used to determine if the indicator in the tool's checklist does meet or not the EGD criterion. If the indicator meets the criterion, the answer is “True”, and if the indicator does not, the answer is “False”. The weight of indicators was neglected. To apply this approach, a grid of checkboxes on spreadsheets, with the indicators in rows and the EGD criteria in columns was created (Fig. 5, Fig. 6). If a box is marked, it signifies that the indicator is directly or indirectly connected to the criterion, so the response is “True”. Whilst an empty box means that the answer is “False” and there is no connection between the indicator and the criterion. When checking the boxes, a vertical histogram is created automatically in the side column of the grid, displaying how many criteria of the EGD the indicator has responded to (Fig. 6, Annexes 2, 3, and 4). All kinds of buildings are considered while filling the checkboxes; the building could be residential, educational, industrial, food warehouses, offices, etc. As an example, when evaluating the sustainability of a food warehouse, the indicator “flooding” is in a direct link with the food system. Therefore, the seventh criteria of the EGD which is “Farm to fork” is concerned by the indicator “flooding”. But if the building to be assessed is an office, in this case, there is no link between “flooding” in an office building with the criterion farm to fork in EGD. Since one type of building could make the indicator meet the criterion, the box is marked as a positive answer. To better interpret the results of this Boolean analysis, mathematical calculations using MATLAB were carried out after converting the grids into tables with binary numbers in the program. A value of 1 reflects a checked box, while a value of 0 reflects an unchecked box. The MATLAB code consisted of counting the sum of boxes containing the number one, first in the whole grid (equation (1)) and then for each column independently (Equation (2)). The findings obtained represent two aspects: firstly, how much the tools respond to the entire EGD criteria, and secondly, the tool's response rate to each criterion of EGD individually. An example of the MATLAB code and matrix are presented in Annex 1.

(1) $$f\left(c\right)=\frac{\sum_{i=1}^{n}\sum_{j=1}^{8}c_{ij}}{8n}\times 100$$

(2) $$f_{j}\left(c\right)=\frac{\sum_{i=1}^{n}c_{i\dot{j}}}{n}\times 100$$

With:$\left\{ \begin{array}{c} C\;is\;the\;cell\;in\;the\;matrix\;\left(the\;boxes\;in\;the\;grid\right)\\ i\;is\;the\;number\;of\;columns\;\left(EGD\;criteria\right)\\ j\;is\;the\;number\;of\;rows\;\left(the\;number\;of\;indicators\right) \end{array}\right.$.

### 2.3 Fuzzy logic analysis

**Fig. 7:**
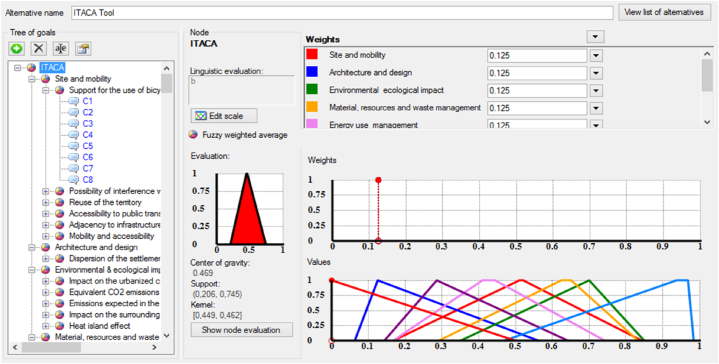
FuzzMe interface with the tree nodes on the left, and weights and results on the right.

**Fig. 8:**
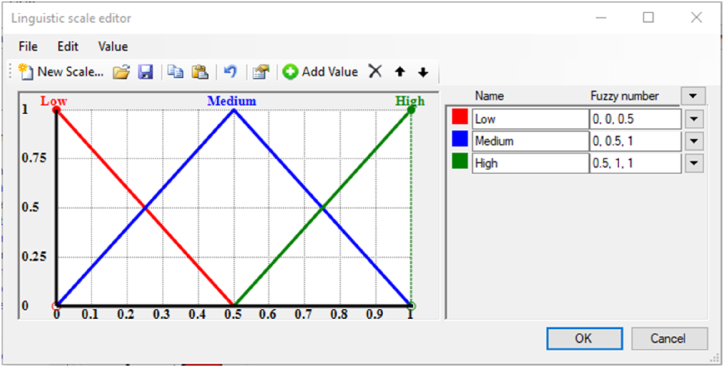
Scale selection interface.

The Fuzzy Logic theory has been shown as a very effective method for addressing uncertainty and ambiguity in Multiple Criteria Decision Analysis (MCDA) [[57], [58], [59]]. The “FuzzME” software was created based on this concept [60,61]. Since filling the grid of checkboxes about whether the indicators are meeting the EGD criteria or not is surrounded by uncertainty related to authors judgement, and due to the ease of use and the results reliability of this software, “FuzzMe” was selected to achieve the task. The data are represented by a hierarchical structure known as an “objectives tree”, where the root (the node) represents the final goal while the branches present the sub-goals (sub-nodes) of the evaluation process. These subgoal contributes to the fulfillment of the goal by a value within the interval [0,1]. Each branch could also be subdivided into several levels of ramifications that are either qualitative or quantitative. In the case of our study, each tool represents the node (root of the tree), and the sub-nodes (the branches) are the tools categories. The ramifications serve as indicators, and each of these indicators is connected to eight qualitative categories that present the eight EGD criteria (Fig. 7). The response of one indicator to an EGD criterion is evaluated by three ranges: (Low) for the triangular interval [0, 0, 0.5], (Medium) for the interval [0, 0.5, 1], and (high) for the interval [0.5, 1, 1], meaning respectively, low, medium, and high response to the criterion in question (Fig. 8).

The intersection of the several intervals reflects the ambiguity in predicting the indicator's response to the criterion, or the wide interpretation of the criterion. Thus, this methodology's use of fuzzy logic increases the reliability of the findings, in terms of choosing the tool that best aligns with the EGD criteria. Fig. 7 represents the interface of the software with the tree structure of the tool on the left, weights, and results on the right side. The node and sub-nodes type in FuzzMe is the “Fuzzy weighted average”, while the EGD criteria’ type is “qualitative criteria”. The weights of the indicators were equally normalized; it means that all indicators belonging to the same cluster have equal influence in it assuming that all weights are equal to the value 1. Only the level of response to the sustainability criteria of EGD were considered.

## 3 Results

### 3.1 Boolean analysis results

**Fig. 9:**
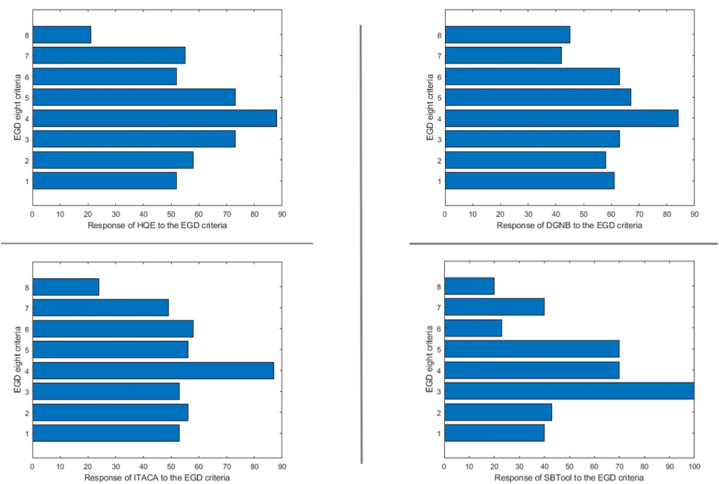
Diagrams presenting the overall response of each tool to the (European Green Deal) EGD eight criteria. (For interpretation of the references to colour in this figure legend, the reader is referred to the Web version of this article.)

**Fig. 10:**
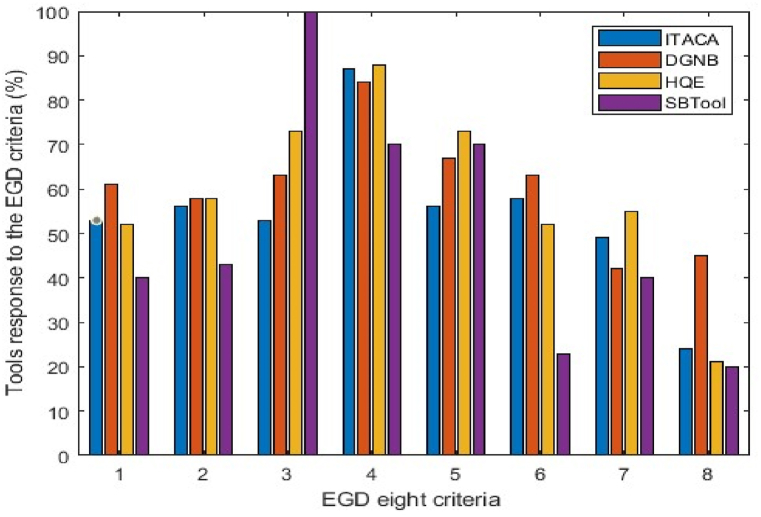
Histogram comparing the indicators compliance with each of the EGD criteria.

We converted the answers of the indicators to the EGD criteria into binary values, which enabled us to analyze the data using MATLAB. Initially, we computed the proportion of affirmative responses within the grid, namely the extent to which the indicators align with the EGD targets. Table 2 displays the acquired results, while Fig. 9 demonstrates the response of each tool to the eight criteria. The displayed graphs in this figure were combined to create the histogram depicted in Fig. 10. The results indicate that the differentiation of the indicators' responses is not consistent across all criteria. The tools indicators are mostly relevant to criterion three, which is “mobilizing industry for a clean and circular economy», having a range of effectiveness from 53 % to 100 %. Criteria one (Increasing the EU's climate ambition for 2030 and 2050) and Five (A zero-pollution ambition for a toxic-free environment) follows criterion three, in terms of relevance, with respective ranges [40, 61] and [56, 73]. On the other hand, criterion eight (Accelerating the shift to sustainable and smart mobility) is by far the least represented varying from 20 to 45 %. Criteria two (Supplying clean, affordable, and secure energy) and six (preserving and restoring ecosystems and biodiversity) demonstrate a high degree of similarity in terms of their relevance to the tools, with an average range of 50 % for both. The criterion four is spread over a range from 70 % to 88%).

**Table 2:** Percentage of meeting each criterion of EGD by ITACA, DGNB, SBTool, HQE: The maximum value in each column is highlighted.

	C1	C2	C3	C4	C5	C6	C7	C8
ITACA	53	56	53	87	56	58	49	24
DGNB	61	58	63	84	67	63	42	45
HQE	52	58	73	88	73	52	55	21
SBTOOL	40	43	100	70	70	23	40	20

The graph in Fig. 10 also shows that some bars are particularly higher or lower in comparison to the rest of the tools. For example, SBTool responds to the third criterion with a percentage of 100 %, while for the rest of the tools the response is around 60 %. On the other hand, SBTool has by far the weakest response to criterion 6 in comparison to HQE, DGNB, and ITACA.

### 3.2 Fuzzy logic analysis results

**Fig. 11:**
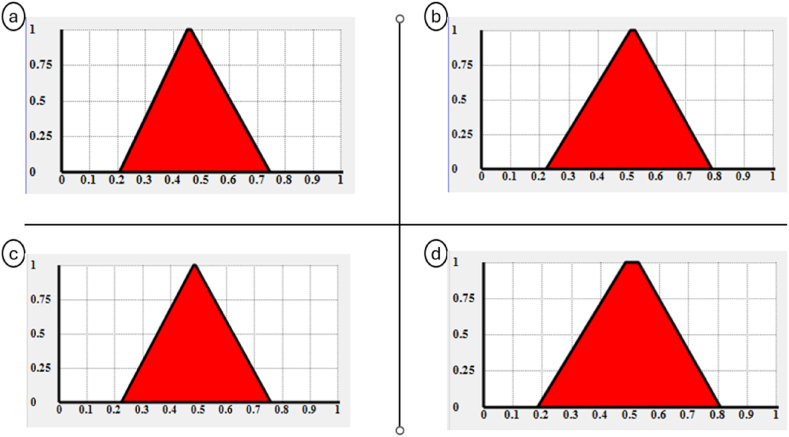
Graphs presenting the results obtained from the Fuzzy logic analysis of: a. ITACA b. DGNB c. SBTool d. HQE.

The findings of the FuzzMe research indicate that all four tools have more or less a close output in meeting the EGD criteria. The assessment results of the nodes are displayed as geometric shapes with a support and a kernel. The center of gravity and uncertainty level of the evaluation can be inferred from this shape (Fig. 11). In fact, the best result corresponds to the tool having the smaller support, and the wider closest to 1 kernel. Also, good results are interpreted by a low uncertainty and a higher value of gravity center.

**Fig. 12:**
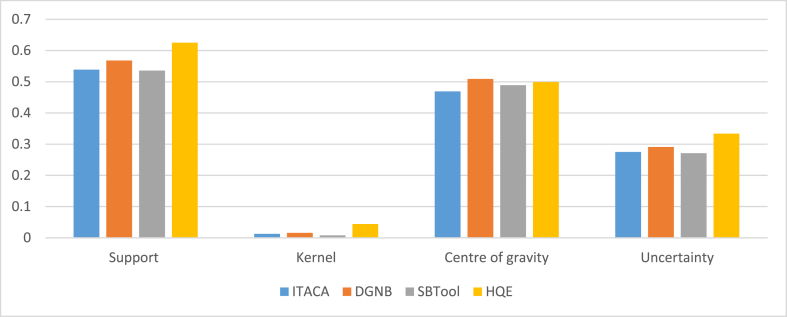
Comparison between node evaluation results.

Table 3 presents the results obtained for the four tools. The DGNB tool and HQE have the highest overall performance, followed by SBTool, and ITACA. While the results are similar, the French tool exhibits a broader kernel but with a significant level of uncertainty and support range. On the other side, DGNB has the highest center of gravity (Fig. 12).

**Table 3:** Nodes evaluation results of ITACA, DGNB, SBTool and HQE: The best results are highlighted.

	ITACA	DGNB	SBTool	HQE
Support	**[0.206, 0.745]**	**[0.221, 0.789]**	**[0.223, 0.759]**	**[0.183, 0.808]**
Kernel	**[0.449, 0.462]**	**[0.51, 0.526]**	**[0.481, 0.489]**	**[0.484, 0.528]**
Centre of gravity	**0.469**	**0.509**	**0.489**	**0.499**
Uncertainty	**0.275**	**0.291**	**0.271**	**0.334**

## 4 Discussion

This work presents a method for evaluating and selecting the best tool aligning with the sustainability and carbon neutrality plan adopted by the European Union community. The construction industry is heavily impacting the environment, and finding solutions to minimize these impacts has been mandatory. Among the most employed green buildings assessment tools used in Europe, this work helped pick the most relevant tool regarding the EGD criteria, a roadmap that is being followed in the region to achieve environmental neutrality. Therefore, the best performant estimated tool could be used as reference for improving the other existing tools or the building evaluation protocols that will be developed in the future. This study analyzed and took into consideration particularly every single indicator in the ITACA, DGNB, SBTool, and HQE checklists, that were already selected for their use and application within the same location. Moreover, the analysis was based on an existent roadmap towards sustainability and carbon neutrality. In fact, the similarities in the tools' checklists made it challenging to do the task. Therefore, the combination of Boolean and fuzzy logic techniques of Multi-Criteria Decision Making helped achieve the aim of this study. The utilization of MATLAB and FuzzMe programs facilitated the attainment of more conclusive results by showing that the DGNB tool is the most suitable for tracking the roadmap of the EGD strategy. Our findings indicate that the tool assigns balanced significance to all clusters, except the architectural and design indicator, which is considered by the other tools. A similar deduction was obtained by Sánchez Cordero, A. et al. (2019) [62] in a critical comparison between several green rating systems including DGNB and HQE putting the European Level(s) policy as reference, although the study didn't encompass ITACA or SBTool and didn't take into consideration the location where the compared tools were developed. Research elaborated by Mattoni B. et al. (2018) [26] noticed the importance of more developing the ITACA tool to be more performant in comparison to other international tools such as LEED or BREEAM. A work of Bernardi E. et al., in 2017 mentioned that DGNB and HQE are better to cover all the life cycle of the buildings and the urban planning project than SBTool. However, these aspects were developed in the latest version of SBTool. It is crucial to observe that the criteria of the EGD are specifically determined for certain aims, which explains why it is not possible for indicators to fulfill all eight criteria in many cases. The HQE tool presents very high efficiency overall. However, there is no balance in giving the same importance to all criteria, especially for energy and mobility. The SBTool is at an average level in comparison to the rest of the tools. This is very understandable since this tool is the basis on which the three other tools were built, then it was sculptured depending on the architectural and constructional specificities of each country. The ITACA protocol shows comparable results shape to the DGNB tool, and it shows an overall good compliance with EGD criteria. Nevertheless, the assessment of energy and environmental impacts by means of this tool does not seem to be sufficient in comparison to the rest of the tools. It is important to note that the EGD criteria aim to achieve carbon neutrality and promote environmentally friendly practices in all sectors. On the other hand, the green tools focus on evaluating structures that directly interact with humans, considering social aspects that are not explicitly addressed by the EGD criteria. Stated differently, certain indicators did not match any of the EGD criteria, however, this cannot be interpreted negatively because the tools consider a factor that is essentially absent from the EGD paradigm.

## 5 Conclusion

This study concentrates on the evaluation of the green building assessment tools used in Europe: ITACA, DGNB, SBTool, and HQE in terms of better encompassing the assessment of building sustainability through their indicators. The research underscores the critical necessity of establishing a comprehensive reference framework, akin to the European Green Deal, which can systematically classify and evaluate these assessment protocols in relation to their sustainability impacts. As the methodologies for building evaluations continue to advance, this comparison was challenging. However, the combination of two MCDM showed efficacy in treating these difficulties. The research took into consideration that the evaluated tools developed and applied in the same region, and derived from the same origin which is the SBTool; the reason why it was also included in this comparison. This work helped understanding that significant gaps still to be adjusted in the tools' indicators' lists, particularly in the integration of assessment techniques tailored to specific types of structures. This is notably pertinent for industrial buildings and food-related establishments, which have unique sustainability challenges and requirements. This gap is especially pronounced in the European context, where there is a strong policy-driven focus on achieving high environmental standards and targets, such as environmental neutrality in the near future. The study aims to contribute to more effective and precise sustainability assessment of buildings, ultimately supporting the broader goal of environmental neutrality and sustainable development in the European region and beyond. In this direction, this paper results will be taken into consideration in future research in order to develop, and apply in real life cases, more suitable tools for buildings’ sustainability evaluation, especially in accordance with the European environmental strategies.

## Data availability

Data will be made available on request.

## CRediT authorship contribution statement

**Dorra Kouka:** Writing – review & editing, Writing – original draft, Visualization, Validation, Software, Methodology, Investigation, Formal analysis, Data curation, Conceptualization. **Mariateresa Russo:** Supervision, Resources, Project administration, Funding acquisition. **Francesco Barreca:** Writing – review & editing, Validation, Supervision, Project administration, Methodology, Investigation, Formal analysis, Conceptualization.

## Declaration of competing interest

The authors declare that they have no known competing financial interests or personal relationships that could have appeared to influence the work reported in this paper.
