# Supplementary material for: Building sustainability assessment: A comparison between ITACA, DGNB, HQE and SBTool alignment with the European Green Deal
Source: Heliyon. 2024 Jul 10;10(14):e34478. doi: 10.1016/j.heliyon.2024.e34478 (PMC11298910; doi:10.1016/j.heliyon.2024.e34478)

Annex 1: Presentation of the matrix constituted of binary values that was based on the indicators grid for ITACA (left) and the related Matlab code to extract results (right)


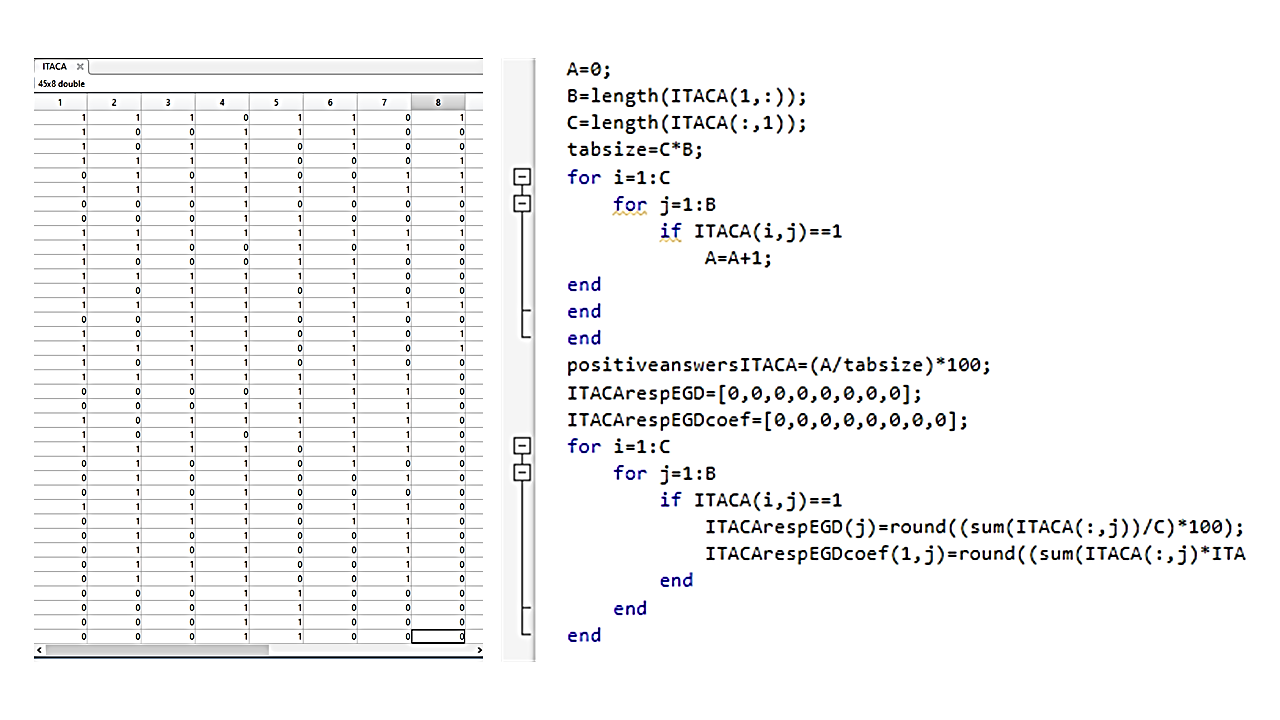


Annex 2: SBTool checklist and grid


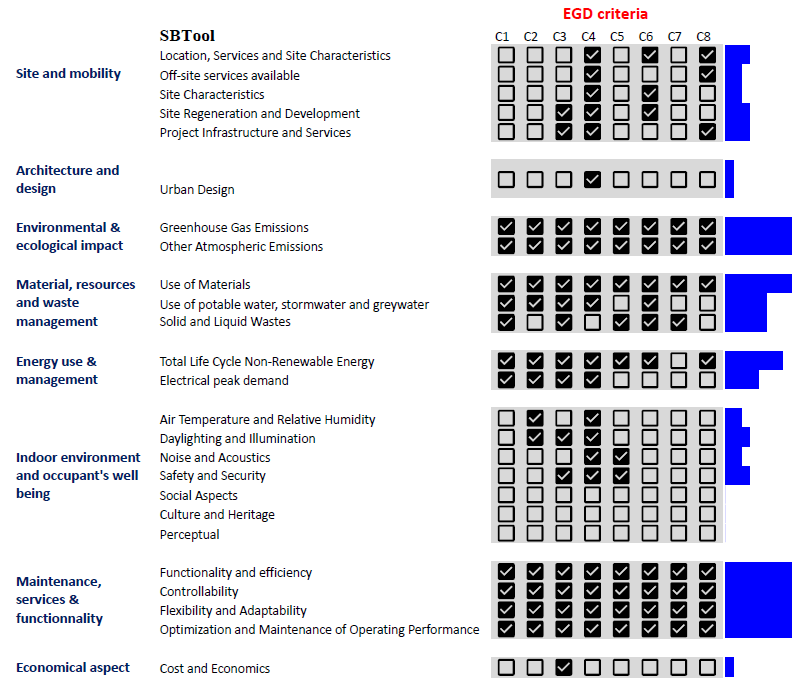


Annex 3: DGNB checklist and grid


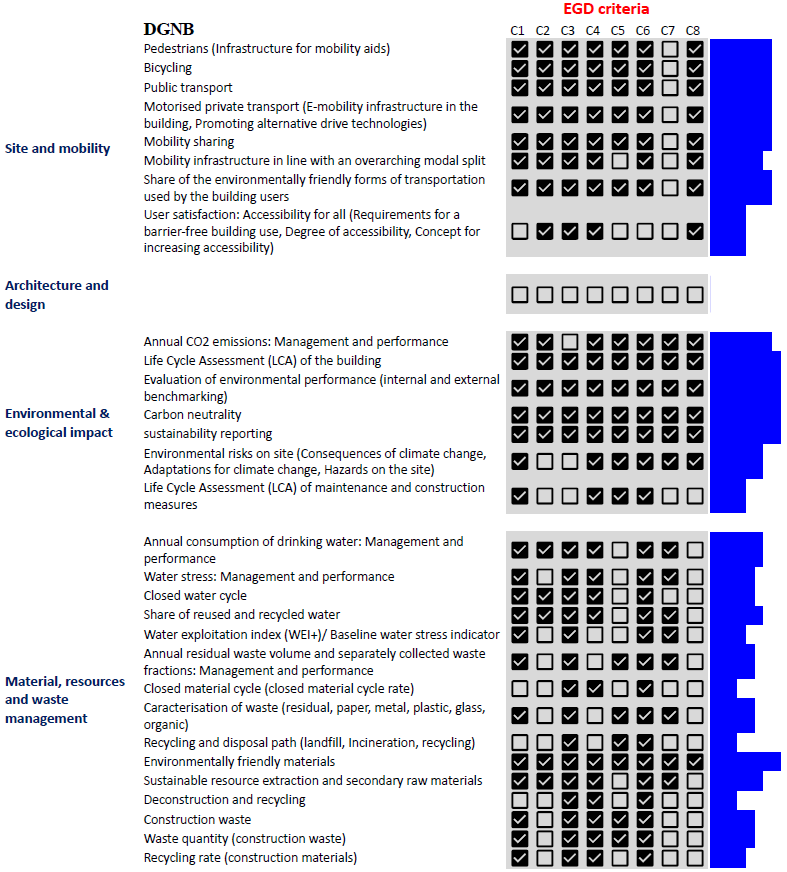


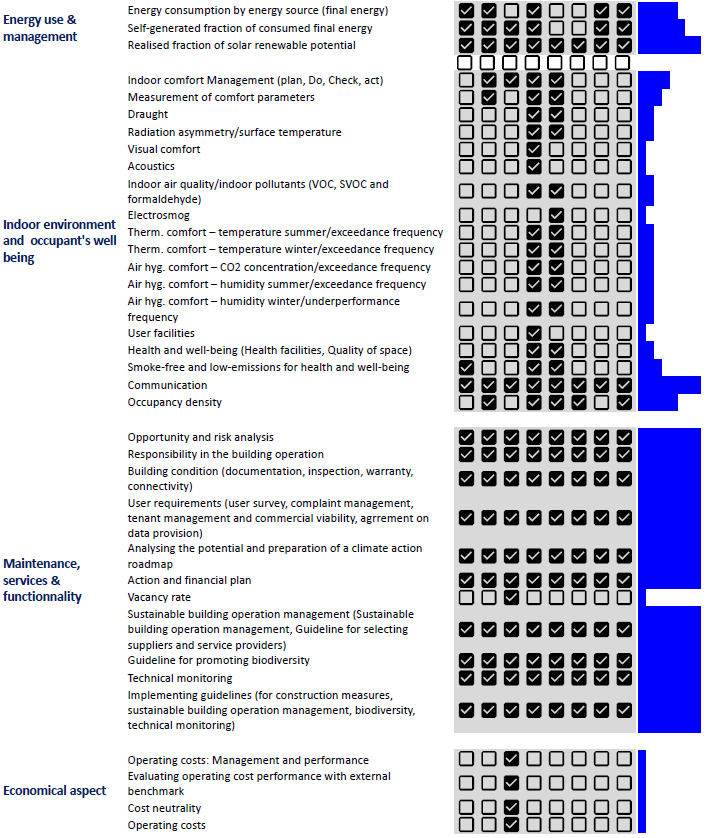


Annex 4: HQE checklist and grid


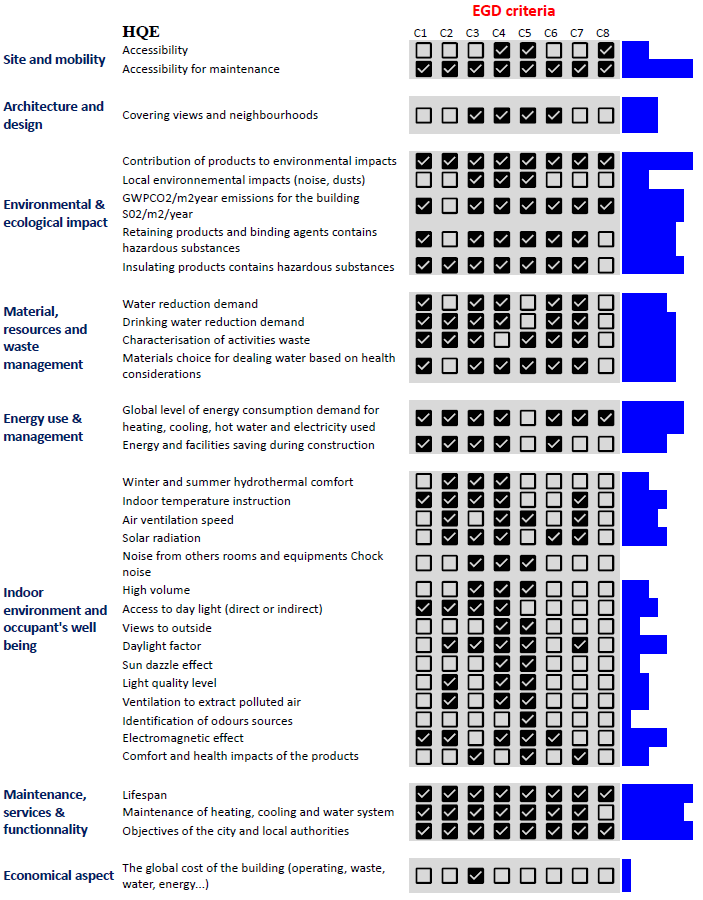

Supplement: Multimedia component 1 [file mmc1.docx]
